# Paradoxical leanness in the imprinting-centre deletion mouse model for Prader–Willi syndrome

**DOI:** 10.1530/JOE-16-0367

**Published:** 2016-11-17

**Authors:** David M Golding, Daniel J Rees, Jennifer R Davies, Dinko Relkovic, Hannah V Furby, Irina A Guschina, Anna L Hopkins, Jeffrey S Davies, James L Resnick, Anthony R Isles, Timothy Wells

**Affiliations:** 1 School of BiosciencesCardiff University, Cardiff, UK; 2 Institute of Life SciencesCollege of Medicine, Swansea University, Swansea, UK; 3 Behavioural Genetics GroupMRC Centre for Neuropsychiatric Genetics and Genomics, Neuroscience and Mental Health Research Institute, Schools of Medicine & Psychology, Cardiff University, Cardiff, UK; 4 Center for Mammalian GeneticsUniversity of Florida, College of Medicine, Gainesville, Florida, USA

**Keywords:** Prader–Willi syndrome, fat mass, thermogenesis, food hoarding

## Abstract

Prader–Willi syndrome (PWS), a neurodevelopmental disorder caused by loss of paternal gene expression from 15q11–q13, is characterised by growth retardation, hyperphagia and obesity. However, as single gene mutation mouse models for this condition display an incomplete spectrum of the PWS phenotype, we have characterised the metabolic impairment in a mouse model for ‘full’ PWS, in which deletion of the imprinting centre (IC) abolishes paternal gene expression from the entire PWS cluster. We show that PWS-IC*^del^* mice displayed postnatal growth retardation, with reduced body weight, hyperghrelinaemia and marked abdominal leanness; proportionate retroperitoneal, epididymal/omental and inguinal white adipose tissue (WAT) weights being reduced by 82%, 84% and 67%, respectively. PWS-IC*^del^* mice also displayed a 48% reduction in proportionate interscapular brown adipose tissue (isBAT) weight with significant ‘beiging’ of abdominal WAT, and a 2°C increase in interscapular surface body temperature. Maintenance of PWS-IC*^del^* mice under thermoneutral conditions (30°C) suppressed the thermogenic activity in PWS-IC*^del^* males, but failed to elevate the abdominal WAT weight, possibly due to a normalisation of caloric intake. Interestingly, PWS-IC*^del^* mice also showed exaggerated food hoarding behaviour with standard and high-fat diets, but despite becoming hyperphagic when switched to a high-fat diet, PWS-IC*^del^* mice failed to gain weight. This evidence indicates that, unlike humans with PWS, loss of paternal gene expression from the PWS cluster in mice results in abdominal leanness. Although reduced subcutaneous insulation may lead to exaggerated heat loss and thermogenesis, abdominal leanness is likely to arise from a reduced lipid storage capacity rather than increased energy utilisation in BAT.

## Introduction

Prader–Willi syndrome (PWS) is caused by a lack of paternal gene expression from the 15q11–q13 imprinting cluster and results from large chromosomal deletions, chromosome 15 maternal uniparental disomy or imprinting-centre (IC) mutations. This disorder is associated with significant metabolic impairment. After severe neonatal hypotonia and a failure to thrive in infancy, subsequent development of hyperphagia and reduced satiety responses result in obesity, unless managed carefully. Indeed, as PWS is the most common syndromal cause of morbid obesity (Butler 1990), elucidating the aetiology of this metabolic disturbance will not only benefit individuals with PWS but also may have wider implications for ameliorating obesity.

Insight into the relationship between the imprinted genes within the PWS cluster and impaired function has been gained by comparing the phenotype of mouse models bearing different PWS-associated mutations. For example, neonatal failure to thrive and early life metabolic impairment are seen in many models (Yang *et al*. 1998, Ge *et al*. 2002, Stefan *et al*. 2005), including a number of single gene knock-out mice (Ding *et al*. 2008, Schaller *et al*. 2010). In contrast, evidence for feeding and metabolic changes in adult mice are inconsistent. For instance, despite being hypophagic (Kozlov *et al*. 2007), mice null for *Magel2* become obese (Bischof *et al*. 2007), whereas mice carrying a deletion of the small nucleolar (sno)RNA *Snord116* show mild hyperphagia, and impaired meal termination, coupled with leanness, even on a high-fat diet (Ding *et al*. 2008).

Given that single gene mutations constitute a rare subset of PWS individuals (Sahoo *et al*. 2008) and display an incomplete phenotypic spectrum (Peters 2008), we have characterised the metabolic impairment in a mouse model for ‘full’ PWS, the PWS-IC*^del^* mouse. IC deletion in this model results in the complete loss of paternal gene expression from the PWS interval (Chamberlain *et al*. 2004), including its expression in the central nervous system (Relkovic *et al*. 2010). Although PWS-IC*^del^* mice suffer considerable postnatal lethality, sufficient survivors have enabled us to demonstrate many of the behavioural aspects of PWS in this model, including reduced locomotor activity and deficits in cognitive function, such as attention and impulse control (Doe *et al*. 2009, Relkovic *et al*. 2010). However, although we have shown that hyperphagia in PWS-IC*^del^* mice is driven primarily by calorie seeking rather than hedonic processes (Davies *et al*. 2015), there has been no systematic characterisation of metabolism in this or any other mouse model for ‘full’ PWS (Bervini & Herzog 2013).

We have therefore characterised the metabolic phenotype of the PWS-IC*^del^* model in this study, quantifying appetitive and consummatory feeding behaviour, lipid profiling and storage, short-term responses to high-fat diet exposure and assessment of thermogenic function.

## Materials and methods

### Animals

The PWS-IC^+/−^ (referred throughout as PWS-IC*^del^*) and wild-type (WT) mice used in this study were bred under the Authority of the Animals (scientific procedures) Act 1986 (UK), with subsequent procedures conforming with the institutional and national guidelines, including those for genetically modified animals, and specifically approved by local ethical review.

PWS-IC*^del^* mice and WT littermates were generated by crossing IC*^del^*-positive males with WT females. Given the nature of the epigenetic regulation of imprinted genes, paternally inherited IC deletion results in a lack of gene expression from the PWS interval. As PWS-IC*^del^* animals on a pure C57BL/6J background suffer severe postnatal lethality, we crossed IC*^del^*-positive males with CD1 females and selectively culled WT littermates (identified on the basis of their increased size 48 h after birth) leaving only 1 or 2 WT pups per litter, as previously described (Chamberlain *et al*. 2004, Davies *et al*. 2015). Animals were weaned at approximately 4 weeks of age and were single-sexed group housed with WT littermates (2–5 animals per cage). This procedure generated the six cohorts of adult mice used below.

All animals were maintained on a 12-h light/darkness cycle (lights on 07:00 h), with *ad libitum* access to water and standard laboratory chow (Rat and Mouse No. 3 Breeding Diet, Special Diet Services Ltd., Witham, Essex, UK, containing 4.2% crude fat; 22.4% crude protein; 4.2% crude fibre; 7.6% crude ash), unless stated otherwise.

### Study 1. Adiposity profile in PWS-IC^del^ mice

After an overnight fast (with water available *ad libitum*), 18-month-old male WT and PWS-IC*^del^* littermate mice were weighed and killed by cervical dislocation. Inguinal, retroperitoneal and epididymal white adipose tissue (WAT) and interscapular brown adipose tissue (isBAT) fat depots and liver were excised, weighed, snap frozen and stored at −70°C for subsequent histological analysis (see below). Left tibiae were excised, and the length was determined with a hand-held micrometre.

### Study 2. Plasma lipid profiles in PWS-IC^del^ mice

Two groups of 16- to 17-month-old female WT and PWS-IC littermate mice were weighed and killed by cervical dislocation after 24 h of total food removal or *ad libitum* feeding (with water available to both groups *ad libitum*). After thoracotomy, blood samples were obtained by cardiac puncture using BD Microtainers (SST Amber Tubes, BD Biosciences, UK), and aliquots of separated plasma were stored at −20°C for the quantification of circulating ghrelin and lipid profiling (see below).

### Study 3. Thermogenesis in PWS-IC^del^ mice

After analysis of BAT histology from the mice in study 1, surface body temperature was measured in 6- to 10-month-old male WT and PWS-IC mice as previously described (Wells *et al*. 2012, Schultz *et al*. 2013). Thermal images obtained using an FLIR A40 camera (FLIR Systems, Boston MA, USA) were analysed by ImageJ to determine surface body temperatures for the head, interscapular, dorso-lumbar and tail root regions and for a region of cage immediately adjacent to the right shoulder.

### Study 4. The effect of suppressing thermogenesis in PWS-IC^del^ mice

To determine the impact of thermogenesis on intra-abdominal adiposity, 6- to 15-month-old male and female PWS-IC*^del^* mice, and WT littermates, were group housed (2–3 mice/cage) at either standard room temperature (20–22°C) or at a thermoneutral ambient temperature (30°C) for 9 weeks. 30°C represents the lower critical temperature in mice, below which non-shivering thermogenesis is induced (Overton 2010). Mice maintained at thermoneutrality were housed in standard cages inside a warming chamber (Scanbur Type 48-VS-IV, Karslunde, Denmark), beginning at room temperature and increasing the temperature by no more than 2°C per day until a stable chamber temperature of 30°C was attained on day 8. Body weight was monitored 3 times/week. In weeks 7–8, mice were individually housed in metabolic cages under standard or thermoneutral ambient temperatures. After 3 days acclimatisation, *ad libitum* food intake was measured on two consecutive days and mean daily food intake calculated. The mice were then returned to their original group cages for the remainder of the 9 weeks, after which mice were weighed and killed by decapitation under isoflurane anaesthesia. Interscapular BAT, inguinal, epididymal/omental and retroperitoneal WAT and liver were excised, weighed, snap frozen and stored at −80°C for subsequent analysis.

### Study 5. Feeding behaviour in PWS-IC^del^ mice

#### Study 5a. *Ad libitum* feeding

Groups of 5- to 9-month-old male and female WT and PWS-IC*^del^* mice were housed individually in metabolic cages. After 5 days acclimatisation, mice were subjected to *ad libitum* feeding with a standard diet (SDS 10% Atwater fuel energy (AFE) Fat, in which energy sources (3.68 kcal/g) were Crude Fat 10%; Crude Protein 20%; Carbohydrate 70% (Special Diet Services, Witham, Essex, UK)) for 1 week, followed by a further 1-week period with a high-fat diet (SDS 45% AFE Fat, in which energy sources (4.54 kcal/g) were: Crude Fat 45% Crude Protein 20%; Carbohydrate 35%). Body weight and food intake were monitored daily, food consumption being normalised to body weight. After the first day of exposure to each diet, the proportion of ‘hoarded’ food was determined by expressing the mass of food in the inner (‘spill’) compartment of the food hopper (Supplementary Fig. 1A and B, see section on supplementary data given at the end of this article) as a percentage of the total remaining diet. The proportion of diet consumed during the darkness and light phases was determined on the fifth day of exposure to each diet. At the end of the dietary manipulations, mice were killed by decapitation under isoflurane anaesthesia and trunk blood was collected. Separated plasma was stored at −20°C prior to subsequent analysis of plasma ghrelin (total) concentration (see below).

#### Study 5b. Post-fast feeding

Group housed 4- to 6-month-old male and female WT and PWS-IC*^del^* mice were subjected to a 16-h overnight fast (17:00–09:00) (with water provided *ad libitum*). Mice were placed individually in normal shoe-box cages under low lighting levels and presented with a pre-weighed pot of wet mash (1 part standard diet (SDS RM3):1 part water). Wet mash was used to overcome the propensity of PWS-IC*^del^* mice to hoard diet pellets and spill powdered diet (Supplementary Fig. 1). Mice were allowed to feed freely for 60 min, the pots being weighed after 30 and 60 min to determine food consumption.

### Tissue analysis

Adipocyte size and hepatic lipid content were quantified as previously described (Davies *et al*. 2009). Samples of retroperitoneal WAT were paraffin-embedded and 5 µm sections were stained with Masson’s Trichrome. Cryostat sections (15 µm) of liver were stained with oil red-O and counterstained with Meyer’s haematoxylin. Digital images (one image per section, three images per mouse) were obtained under light microscopy. Adipocyte size was quantified in cells with transversely sectioned peripheral nuclei (minimum 9 cells per section/3 sections per sample) using ImageJ and hepatic lipid droplets measured using Leica Q-Win. The degree of ‘beiging’ was determined in three random full screen digital images of retroperitoneal WAT. Areas of ‘beiging’ were identified (see Fig. 2a and b), delineated, filled and quantified using ImageJ, the data being expressed as %-‘beiging’ of total field.

Uncoupling protein-1 (Ucp-1) mRNA expression in isBAT and inguinal WAT was quantified as previously described (Wells *et al*. 2012). In brief, total RNA from lysed tissue was purified using the Qiagen RNeasy Mini extraction kit before quantification and assessment of RNA purity. RNA was subsequently reverse transcribed using the Precision nanoScript Reverse Transcription kit (Primerdesign, Southampton, UK), 1 µg RNA being reverse transcribed in 20 µL reactions containing 1 µL oligoDT and 1 µL random decamers (Primerdesign) prior to freeze storing at −20°C. cDNA was diluted 1:10 in dd.H_2_O prior to quantification of *Ucp-1* and *β-actin* cDNA by quantitative real-time PCR amplification using a Bio-Rad IQ5 thermal cycler and Precision 2× qPCR Mastermix reagents (Primerdesign). Oligonucleotide primers for mouse *Ucp-1* were purchased from Primerdesign (*Ucp-1* sense primer, GGTAAAAACAAGATTCATCAACTCTC: *Ucp-1* anti-sense primer, TCGCAGAAAAGAAGCCACAA). PCR thermal cycling conditions included an initial enzyme activation for 10 min at 95°C (1 cycle), followed by 51 cycles of denaturation at 95°C for 15 s and real-time Fluorogenic Data Collection (FDC) at 60°C for 1 min. The final FDCs were acquired at 0.5°C temperature increments between 55°C and 95°C at 10-s intervals (81 cycles). The PCR products were quantified by incorporation of SYBR green into double stranded DNA. Single amplicon identity was verified by melt curve analysis. Individual samples were normalised to the expression of *β-actin* (ACTB Primer mix, Primer Design # ge-SY-6; Batch # 12474). The quantity of cDNA in each reaction was calculated by reference to a standard curve constructed from serial dilutions of cDNA from WT BAT (Wells *et al*. 2012).

### Plasma analysis

Plasma ghrelin (total) concentrations were determined by RIA (Millipore/Linco (IAV: 3.28–8.05%)). Plasma lipids were extracted by the Folch method (Kates 1986). Classes of individual non-polar lipids (triacyglycerols, free fatty acids and sterol esters), and total polar lipids were separated by thin-layer chromatography and quantified by their fatty acid contents. Fatty acid methyl esters were separated on a Clarus 500 gas chromatograph (PerkinElmer) identified by comparing retention times with fatty acid standards (Nu-Chek Prep Inc., Elysian, MN, USA) and quantified against an internal pentadecanoate standard.

### Statistical analysis

Results are expressed as mean ± s.e.m., and differences were established by 1-way and 2-way repeated measures ANOVA and Bonferroni *post hoc* test or unpaired Student’s *t*-test (using GraphPad Prism, GraphPad Software), as indicated in the figure and table legends, with *P* < 0.05 considered significantly different.

## Results

### Growth and endocrine status in PWS-IC^del^ mice

**Figure 1:**
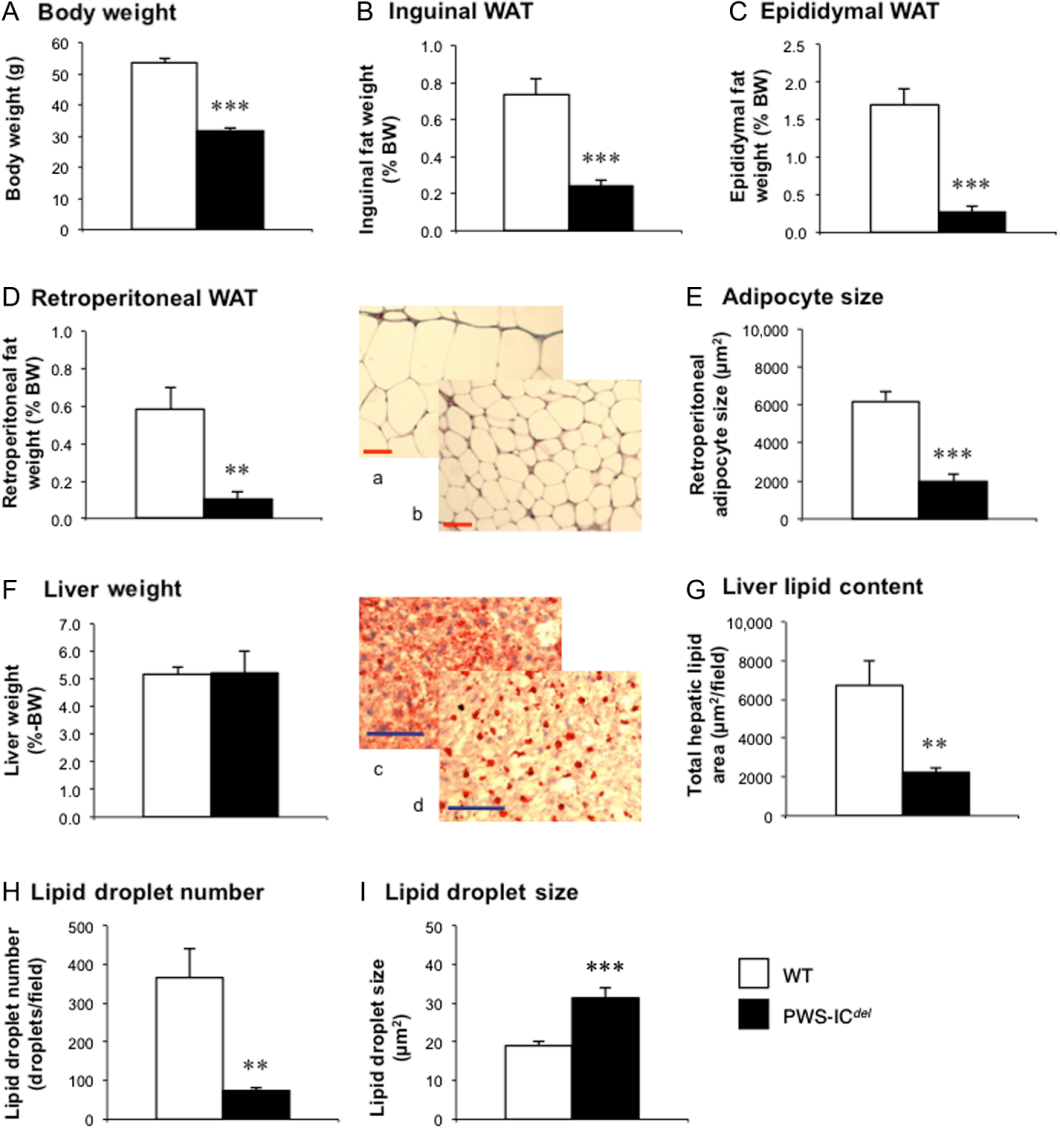
PWS-IC*^del^* mice show profound abdominal leanness and reduced hepatic lipid storage. Quantification of body weight (A), proportionate inguinal (B), epididymal (C) and retroperitoneal (D) WAT, and liver (F) weights in 18-month-old male WT (*n* = 6) and PWS-IC*^del^* (*n* = 6) littermate mice. Retroperitoneal adipocyte size (E) was quantified on digital images from WT (a) and PWS-IC*^del^* (b) mice (scale bar 50 µm). Hepatic lipid content (G), lipid droplet number (H) and size (I) were determined by digital analysis of oil red-O-stained sections in WT (c) and PWS-IC*^del^* (d) mice (scale bar 100 µm). Values shown are mean ± s.e.m., with statistical comparisons performed by Student’s unpaired *t*-test (**P < 0.01; ***P < 0.001 vs WT). A full colour version of this figure is available at http://dx.doi.org/10.1530/JOE-16-0367.

Although neither foetal growth (E18.5 PWS-IC*^del^* foetuses weighed 96% of their WT counterparts (WT: 1.18 ± 0.044 g (*n* = 7); PWS-IC*^del^*: 1.13 ± 0.044 g (*n* = 7); *P* = 0.45)) nor placental weight (WT: 100 ± 6.5 mg (*n* = 7); PWC-IC*^del^*: 92 ± 4.1 mg (*n* = 7); *P* = 0.32) were significantly affected in PWS-IC*^del^* pregnancies, PWS-IC*^del^* mice failed to thrive after 24 h post-partum, leading to increased neonatal mortality. PWS-IC*^del^* mice that survived to adulthood were significantly growth retarded, adult PWS-IC*^del^* males showing a 40% reduction in body weight (Fig. 1A; *P* < 0.001), accompanied by a 7% reduction in tibial length (data not shown; *P* < 0.001). Comparable reductions in body weight were observed in similarly-aged PWS-IC*^del^* females (Table 1).

**Table 1:** The effect of fasting on the plasma lipid profile and circulating ghrelin in female W-T and PWS-IC^*del*^ littermate mice.

	**W-T female**		**PWC-IC^*del*^ female**	
	**Fed** (*n* = 9)	**Fasted** (*n* = 9)	**Fed** (*n* = 8)	**Fasted** (*n* = 4)
Body weight (g)	43.1 ± 3.0	42.2 ± 2.6	26.2 ± 0.7^aaa^	22.9 ± 1.1^ccc^
Total polar lipids (µg/µL)	0.313 ± 0.021	0.366 ± 0.043	0.247 ± 0.014	0.269 ± 0.040
Free fatty acids (µg/µL)	0.045 ± 0.004	0.072 ± 0.013	0.049 ± 0.017	0.069 ± 0.021
Triacylglycerol (µg/µL)	0.191 ± 0.026	0.105 ± 0.023	0.128 ± 0.031	0.085 ± 0.042
S1 sterol esters (µg/µL)	0.036 ± 0.005	0.057 ± 0.011	0.029 ± 0.005	0.025 ± 0.009
S2 sterol esters (µg/µL)	0.058 ± 0.004	0.058 ± 0.010	0.045 ± 0.007	0.030 ± 0.009
Plasma [ghrelin (total)] (ng/mL)	9.82 ± 2.24	54.94 ± 10.03^aa^	13.12 ± 1.95	62.86 ± 15.43^bb^

S1, arachidonic acid-enriched sterol esters;

S2, sterol esters with shorter chain fatty acids.

aa *P* < 0.01

aaa *P* < 0.001 vs fed W–T female

bb *P* < 0.01 vs fed PWC-IC^*del*^ female

ccc *P* < 0.001 vs fasted W-T female.

### Study 1. Adiposity profile in PWS-IC^del^ mice

However, in contrast to humans with PWS, PWS-IC*^del^* mice were surprisingly lean, proportionate inguinal (Fig. 1B), epididymal (Fig. 1C) and retroperitoneal (Fig. 1D) WAT weights being reduced by 67% (*P* < 0.01), 84% (*P* < 0.001) and 82% (*P* < 0.01), respectively, and reflected in a 69% reduction in adipocyte size (Fig. 1E and inset pictures a and b; *P* < 0.001). Although proportionate liver weight was unaffected (Fig. 1F), hepatic lipid storage in PWS-IC*^del^* mice was disrupted. In contrast to the diffuse lipid staining in WT livers (Fig. 1c inset), PWS-IC*^del^* livers showed punctate staining (Fig. 1d inset), with fewer (80% of WT (Fig. 1H; *P* < 0.01)), larger (increased by 66% (Fig. 1I; *P* < 0.001)) lipid droplets, with total lipid content reduced by 67% (Fig. 1G; *P* < 0.01).

### Study 2. Plasma lipid profiles in PWS-IC^del^ mice

We therefore investigated whether the reduction in WAT mass was due to impaired lipid handling. Although circulating total polar lipid in fed PWS-IC*^del^* mice was only 79% of that in WT littermates (*P* > 0.05; Table 1), none of the markers of dietary lipid intake (S1 and S2 sterol esters), hepatic lipid output (triacylglycerol: TAG) or WAT lipid output (free fatty acids: FFAs) were significantly different. Similarly, the effect of fasting on circulating lipids was comparable in WT and PWS-IC*^del^* mice (Table 1). As shown previously (Davies *et al*. 2015), circulating ghrelin (total) in PWS-IC*^del^* females was 134% of that in female WT littermates (Table 1), but was regulated appropriately after the 24-h fast.

### Study 3. Thermogenesis in PWS-IC^del^ mice

**Figure 2:**
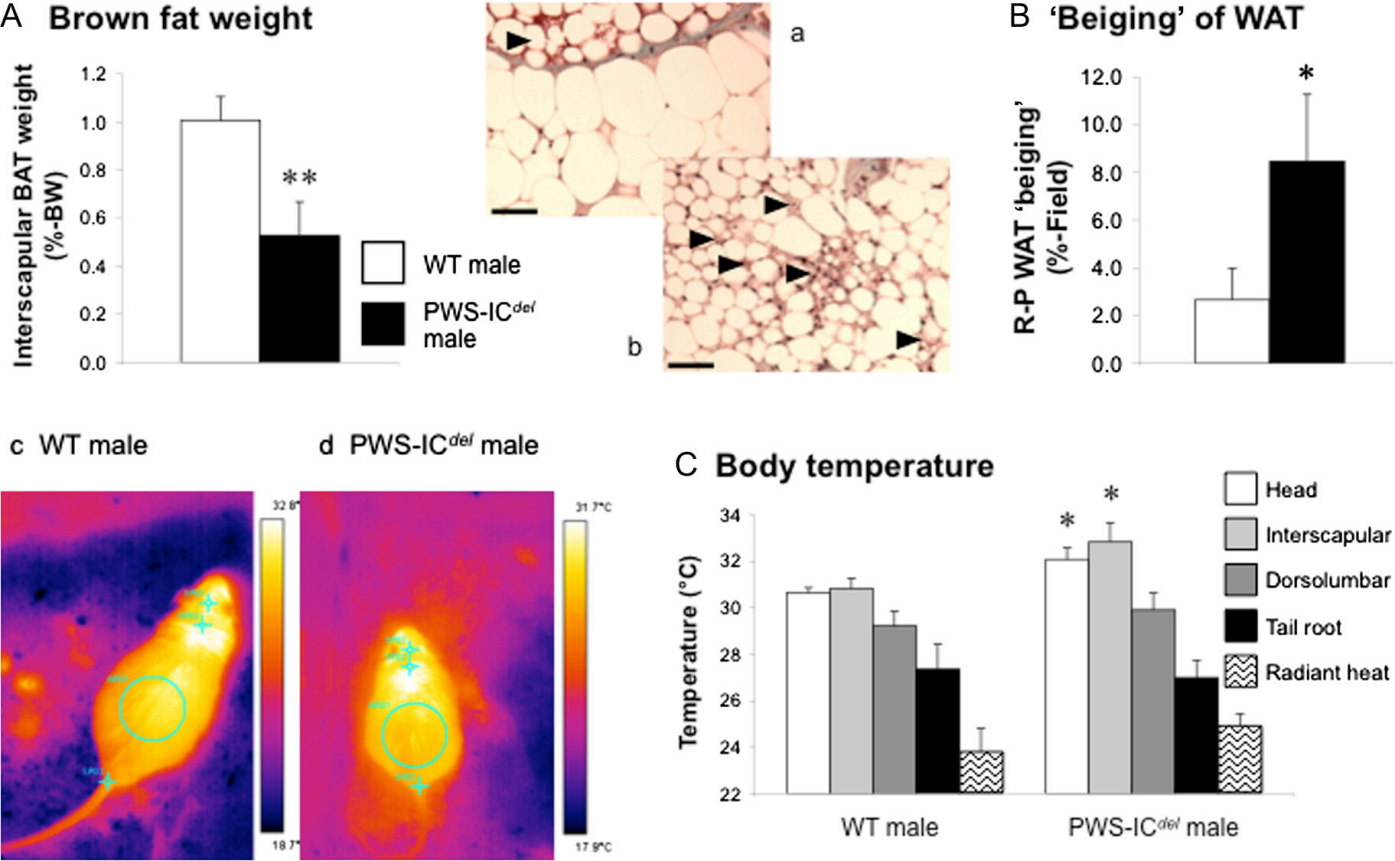
PWS-IC*^del^* mice show enhanced thermogenesis. Quantification of BAT mass (A) and ‘beiging’ in retroperitoneal (R-P) WAT (inset pictures a (WT) and b (PWS-IC*^del^*) (arrowheads: areas of ‘beige’ or ‘brite’ adipose tissue; scale bars: 50 µm)) (B) in 18-month-old male WT and PWS-IC*^del^* littermate mice and determination of surface body temperatures (head, interscapular, dorsolumbar and tail root regions and an estimation of heat loss) from thermal images (inset pictures c and d) taken of 5- to 9-month-old male WT and PWS-IC*^del^* mice (C). Data shown are mean ± s.e.m. (*n* = 6 for all groups), with statistical comparisons made with either Student’s *t*-test (A and B; *P < 0.05, **P < 0.01) or 1-way ANOVA and Bonferroni’s selected pairs *post hoc* test (C; *P < 0.05). A full colour version of this figure is available at http://dx.doi.org/10.1530/JOE-16-0367.

We subsequently investigated the thermogenic activity in PWS-IC*^del^* mice. Proportionate isBAT weight was reduced by 48% in PWS-IC*^del^* mice (Fig. 2A; *P* < 0.01), consistent with elevated thermogenesis. Similarly, retroperitoneal WAT (Fig. 2 pictures a and b) showed a four-fold elevation in the degree of differentiation to ‘beige’ or ‘brite’ adipose tissue (Fig. 2B; *P* < 0.05). Thermal imaging revealed that surface temperatures of the head and interscapular regions were increased in PWS-IC*^del^* mice by 1.4°C and 2.0°C, respectively (Fig. 2C and pictures c and d; *P* < 0.05), whereas dorsolumbar tail root and radiant temperatures were not significantly affected (Fig. 2C).

### Study 4. The effect of suppressing thermogenesis in PWS-IC^del^ mice

**Figure 3:**
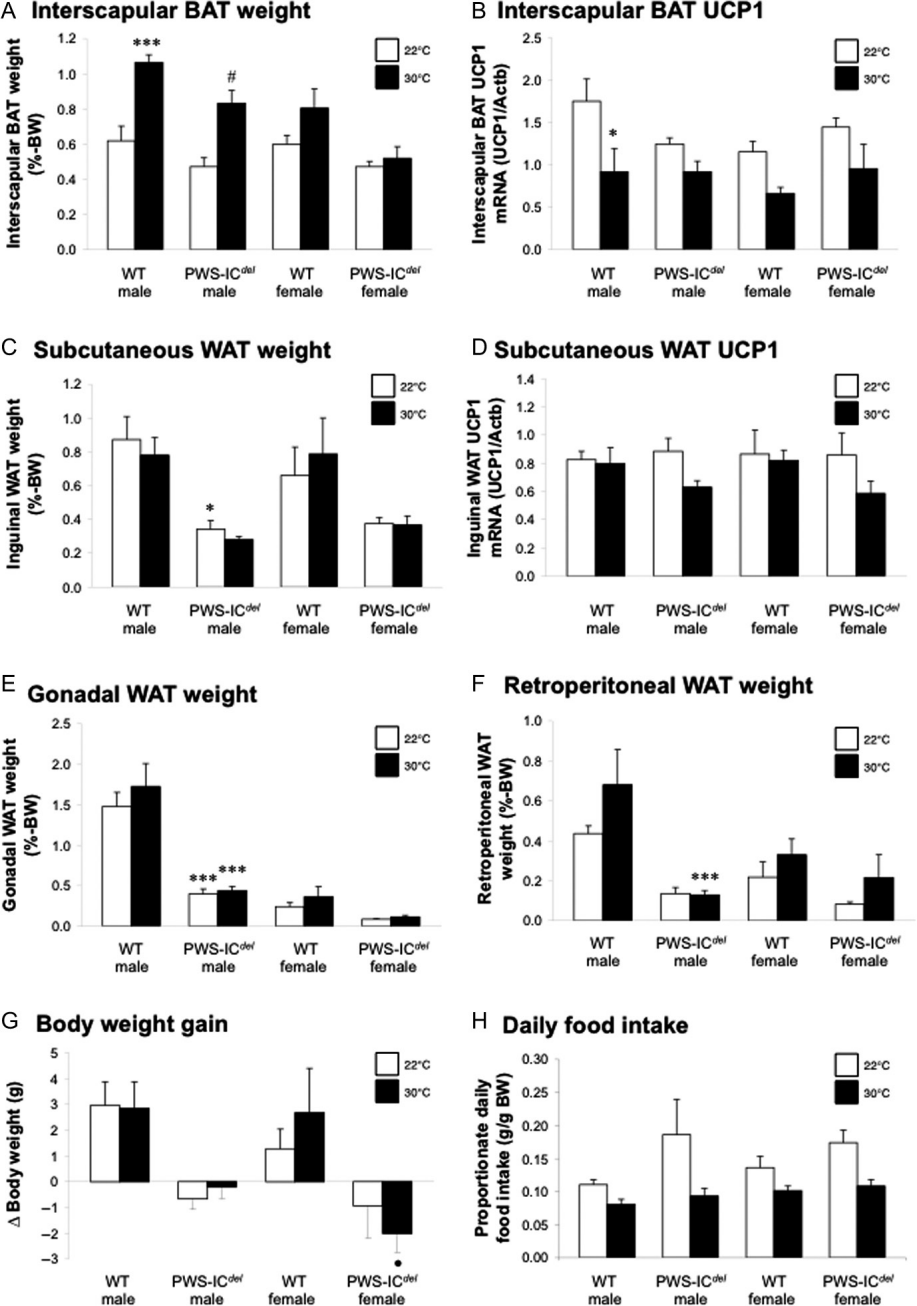
Thermoneutrality suppresses thermogenesis and hyperphagia in PWS-IC*^del^* mice. Quantification of interscapular BAT mass (A) and *Ucp-1* mRNA expression (B), inguinal WAT mass (C) and *Ucp-1* mRNA expression (D), epididymal/omental (E) and retroperitoneal (R-P: F) WAT mass, body weight gain (G) and daily food intake (H) in 6- to 14-month-old male and female WT and PWS-IC*^del^* littermate mice maintained at either standard room temperature (20–22°C; white bars) or thermoneutrality (30°C; black bars) for 9 weeks. Data shown are mean ± s.e.m. (*n* = 5 (WT and PWS-IC*^del^* males at 30°C) and 6 (all other groups)), with statistical comparisons made with 1-way ANOVA and Bonferroni’s selected pairs *post hoc* test (*P < 0.05, ***P < 0.001 vs WT male (room temperature); ^#^*P* < 0.05 vs PWS-IC*^del^* male (at room temperature); ^•^*P* < 0.05 vs WT female (at thermoneutrality)).

To establish whether the increased energy demands of elevated thermogenesis account for the lean phenotype, PWS-IC*^del^* mice and WT littermates, were maintained under thermoneutral conditions (30°C). After 9 weeks, proportionate isBAT weight in WT and PWS-IC*^del^* males was elevated by 72% and 76%, respectively (Fig. 3A), indicating reduced lipid utilisation; these changes being less pronounced in WT and PWS-IC*^del^* females. Thermoneutrality halved *Ucp-1* mRNA expression, the classic marker of thermogenic activity, in WT males (Fig. 3B), but this effect was not significant in PWS-IC*^del^* animals (*P* = 0.053 (males); *P* = 0.183 (females)). Despite suppressed thermogenesis, proportionate WAT mass (total: data not shown, or individual depots: Fig. 3C, E and F) was not increased. For example, although proportionate retroperitoneal WAT mass in WT males at thermoneutrality was 156% of that in corresponding mice at room temperature, these means were not significantly different (Fig. 3E; *P* = 0.09), whereas for male PWS-IC*^del^* mice at thermoneutrality, retroperitoneal WAT mass was 97% of that in PWS-IC*^del^* mice at room tempera­ture. In addition, thermoneutrality had no significant effect on WAT *Ucp-1* mRNA expression (Fig. 3D). However, thermoneutrality reduced liver weight in WT males by 12% (data not shown) suggesting either reduced hepatic liver storage or increased lipid export. Interestingly, suppression of thermogenesis failed to increase body weight gain in any of the groups (Fig. 3G), although WT females at thermoneutrality showed a transient increase in weight gain between days 11 and 15 (*P* < 0.05; data not shown). This lack of weight gain may arise from the suppressive influence of thermoneutrality on daily food intake (Fig. 3H), mean proportionate daily food intake in WT males, PWS-IC*^del^* males, WT females and PWS-IC*^del^* females at thermoneutrality being 73%, 50%, 74% and 62% of that in mice housed at room temperature. Proportionate hyperphagia in PWS-IC*^del^* mice was completely abolished at thermoneutrality (Fig. 3H).

### Study 5. Feeding behaviour in PWS-IC^del^ mice

#### Study 5a. *Ad libitum* feeding

**Figure 4:**
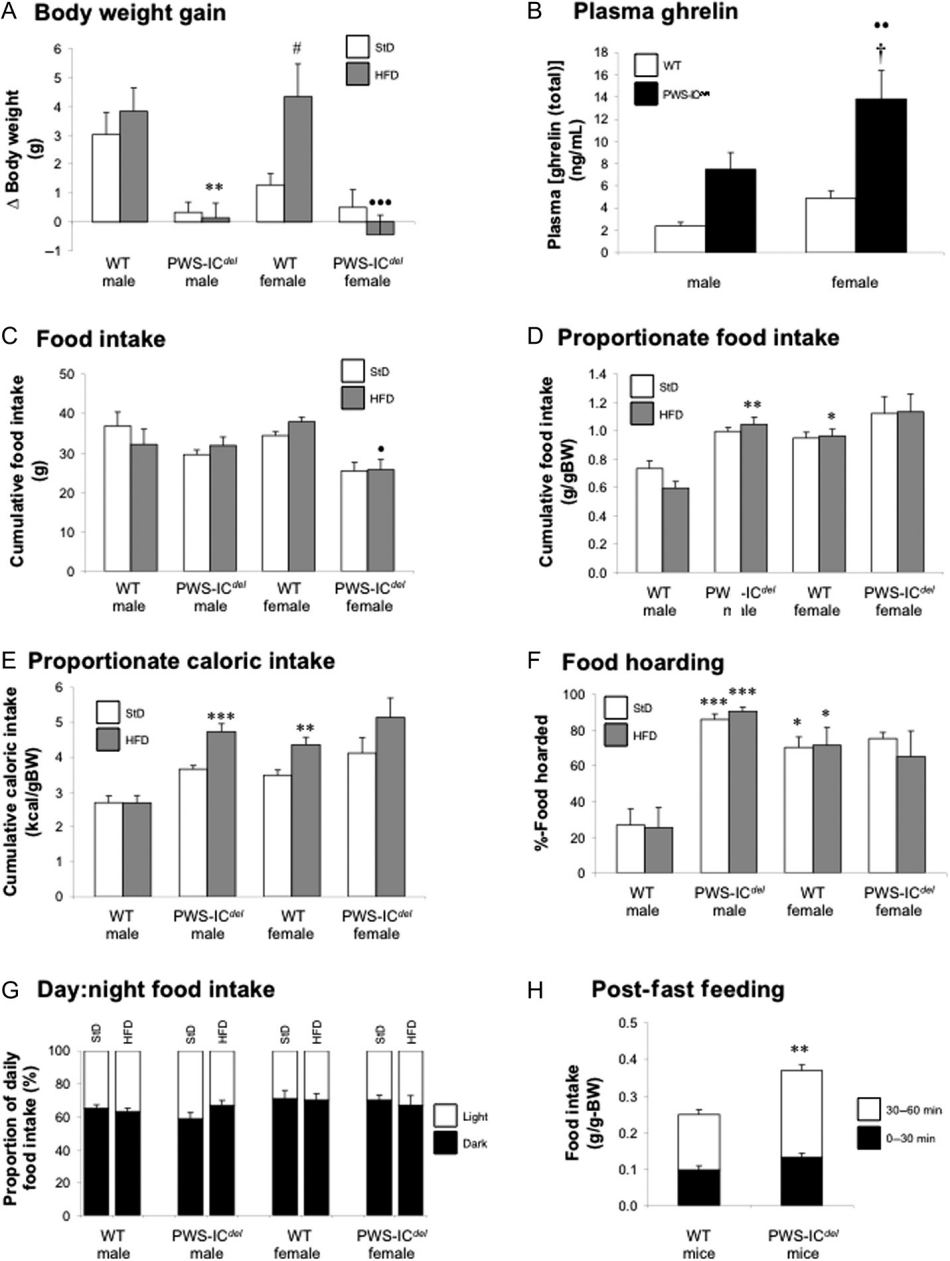
PWS-IC*^del^* mice show hyperghrelinaemia and disproportionately high food intake with exaggerated food hoarding behaviour and impaired meal termination. Analysis of body weight gain (A), terminal plasma ghrelin (total) concentration (B), food intake (C), weight-corrected food intake (D), weight-corrected caloric intake (E), food hoarding behaviour (F) and day:night feeding (G) in 5- to 9-month-old male and female WT and PWS-IC*^del^* littermate mice when fed for 1 week with standard (StD) and high-fat (HFD) diets. Post-fast feeding (H) was determined in 4- to 6-month-old WT and PWS-IC*^del^* littermate mice for 0–30 and 30–60 min after a 16-h overnight fast. Data shown are mean ± s.e.m. (*n* = 6 for all groups), with statistical comparisons made with 1-way ANOVA (A–G) or repeated measures 2-way ANOVA (H) and Bonferroni’s selected pairs *post hoc* test (*P < 0.05, **P < 0.01, ***P < 0.001 vs WT male (same diet); ^#^*P* < 0.05 vs WT female (standard diet); ^•^*P* < 0.05; ^••^*P* < 0.01; ^•••^*P* < 0.001 vs WT female (same diet); ^†^*P* < 0.05 vs PWS-IC*^del^* male (same diet)).

We subsequently examined feeding behaviour in PWS-IC*^del^* mice at normal ambient temperature. Food intake was measured in male and female WT and PWC-IC^*del*^ mice, maintained for 1 week on standard and high-fat diets. Male and female WT mice ate roughly similar amounts of both diets (Fig. 4C). Although PWS-IC*^del^* females consumed 32% less of the high-fat diet (compared to WT females; *P* < 0.05; Fig. 4C), when corrected for body weight, PWS-IC*^del^* males consumed 76% more of the high-fat diet (compared to WT males; *P* < 0.01; Fig. 4D). Interestingly, although WT males maintained a constant proportionate caloric intake irrespective of which diet they were eating, PWS-IC*^del^* males were susceptible to overconsumption when eating the high-fat diet (Fig. 4E). Similar patterns of caloric intake were observed in WT and PWS-IC*^del^* females (Fig. 4E). Adiposity is influenced by the timing of food consumption (Glad *et al*. 2011), but day:night food intake ratios were not significantly different between WT and PWS-IC*^del^* mice (Fig. 4G).

During this experiment, a marked difference in appetitive behaviour became apparent. When presented with refilled diet hoppers PWS-IC*^del^* mice immediately began moving the diet from the outer ‘fill’ compartment to the inner ‘spill’ compartment (Supplementary Fig. 1A and B). When quantifying the amount of diet in the ‘spill’ compartment as a percentage of the remaining diet, PWS-IC*^del^* males were found to ‘hoard’ more than 3-times as much diet as WT males (*P* < 0.001; Fig. 4F and Supplementary Fig. 1A). This behaviour was prominent with both standard and high-fat diets, PWS-IC*^del^* males hoarding 86–91% of the remaining diet. Interestingly, this pattern of hoarding in PWS-IC*^del^* males was broadly similar to that in both WT and PWS-IC*^del^* females (Fig. 4F).

Although constrained by the short period of monitoring, it is particularly striking that although PWS-IC*^del^* mice consumed most calories when maintained on the high-fat diet, they not only failed to gain weight but also gained markedly less weight than their respective WT littermates (*P* < 0.01; Fig. 4A). Analysis of terminal blood samples revealed that mean circulating ghrelin (total) was tripled in PWS-IC*^del^* mice in comparison to their sex-matched littermates (Fig. 4B), although this was not statistically significant in PWS-IC*^del^* males (*P* = 0.116).

#### Study 5b. Post-fast feeding

As PWS-IC*^del^* mice show proportionate hyperphagia, we investigated individual meal consumption patterns after an overnight fast. When adjusted for body weight, PWS-IC*^del^* mice consumed 48% more diet than WT littermates (Fig. 4H; repeated measures ANOVA, main effect of GENOTYPE *F*_1,11_ = 6.01, *P* = 0.032). This was due mainly to continued consumption after 30 min as evidenced by a significant interaction with the time of measurement (*F*_1,11_ = 13.43, *P* = 0.004).

## Discussion

Loss of paternal gene expression from the imprinted gene cluster on human chromosome 15q11–q13 impairs neuroendocrine and metabolic function in PWS. However, investigating these impairments in mouse models for PWS has been hampered by high postnatal lethality. We have utilised a mixed genetic background to increase survival, enabling us to characterise metabolic status in a mouse model in which deletion of the homologous PWS-IC results in complete loss of paternal gene expression from the entire PWS locus (Chamberlain *et al*. 2004, Relkovic *et al*. 2010). We now report that, with one notable exception, the metabolic phenotype of PWS is replicated in adult PWS-IC*^del^* mice.

The growth abnormalities normally associated with PWS are recapitulated in PWS-IC*^del^* mice, with reductions in body weight and limb bone length combined with a 40–50% reduction in circulating IGF-1 (unpublished data). As similar growth results have been reported in another ‘full’ genetic mouse model for PWS (Stefan *et al*. 2005, Ding *et al*. 2008), whereas mice with specific deletion of *Magel2* exhibit normal skeletal growth (Bischof *et al*. 2007); this aspect of the PWS phenotype appears to be dependent upon lack of expression of one or more of the other genes within the PWS locus. Indeed, the reduced circulating IGF-1 and elevated GH-releasing hormone mRNA reported in *Snord116del* mice (Qi *et al*. 2016) indicate that loss of *Snord116* expression causes a pituitary-specific deficiency in the GH axis.

Despite growth retardation, PWS-IC*^del^* mice displayed a remarkable reduction in adiposity, affecting intra-abdominal, subcutaneous and intramedullary (data not presented) depots. Although reduced visceral adiposity has been reported in human females with PWS (Goldstone *et al*. 2001, 2004), the profound leanness in the PWS-IC*^del^* mice is unlike the obesity usually associated with this condition in humans. Indeed, the degree of leanness seen in the PWS-IC*^del^* model is considerably greater than the transient reduction in triglycerides in TgPWS mice (Stefan *et al*. 2005), and the mild reduction in fat mass in *Snord116del* mice (Ding *et al*. 2008, Qi *et al*. 2016). Given this surprising result, which corroborates previous reports in younger mice with IC deletions (Yang *et al*. 1998, Chamberlain *et al*. 2004), we examined the aetiology of this leanness, quantifying indices of energy intake, storage and utilisation.

Leanness in PWS-IC*^del^* mice is clearly not due to impaired energy input, with males in particular showing proportionate hyperphagia, especially with a high-fat diet. Similarly, the normal day:night consumption ratios indicate that the altered lipid storage efficiency associated with daytime feeding in nocturnal rodents (Glad *et al*. 2011) does not contribute to the lean phenotype. The hyperphagia seen in PWS-IC*^del^* mice, which is driven by calorie seeking rather than hedonic processes (Davies *et al*. 2015), was even more exaggerated after overnight fasting, where prolonged consumption indicates a failure in meal termination characteristic of PWS. These indices of increased consummatory behaviour are likely to reflect the sustained hyperghrelinaemia observed in both the current and previous studies (Davies *et al*. 2015), but as this is not accompanied by an increase in NPY mRNA expression (unpublished data), the hypothalamic mechanisms underlying proportionate hyperphagia remain to be elucidated. That said, as *Magel2*-null mice show hypophagia (Bischof *et al*. 2007), whereas specific deletion of *Snord116* results in a similar combination of impaired meal termination and leanness (Ding *et al*. 2008), these aspects of the PWS-IC*^del^*mouse phenotype probably result from loss of *Snord116* expression.

When monitoring food intake, it became rapidly apparent that PWS males also displayed a remarkable increase in appetitive behaviour; moving crushed diet from the fill compartment to the spill compartment (Supplementary Fig. 1A and B). When supplied with pelleted diet, individual PWS-IC*^del^* mice from our Cardiff colony displayed increased diet distribution in both home and metabolic cages (Supplementary Fig. 1C), whereas PWS-IC*^del^* mice in a Florida colony packed bedding around high-fat diet pellets in the food hopper (Supplementary Fig. 1D). This hoarding behaviour, previously described in birds, rodents and, in certain circumstances, humans (Deacon 2006, Keen-Rinehart *et al*. 2010) is most prominent in species not readily storing energy in the form of accumulated WAT (Keen-Rinehart *et al*. 2010) and is exaggerated after surgical removal of epididymal WAT (Dailey & Bartness 2008). This implies that the lean phenotype of these mice may be a significant causal factor in this behaviour. Interestingly, our data also indicate that in WT mice, hoarding appears to be a predominantly female behaviour. Therefore, the hyperphagia and hoarding behaviour observed in PWS-IC*^del^* males suggest that loss of paternal gene expression in the PWS locus results in a feminisation of feeding behaviour. Given that the central circuits regulating both consummatory and appetitive feeding behaviours are activated by ghrelin (Nakazato *et al*. 2001, Lawrence *et al*. 2002, Keen-Rhinehart & Bartness 2005), the elevation in food intake and diet hoarding may result from the sustained hyperghrelinaemia seen in this model (Davies *et al*. 2015) and in humans with PWS (Cummings *et al*. 2002, DelParigi *et al*. 2002, Haqq *et al*. 2003, Erdie-Lalena *et al*. 2006, Bizzari *et al*. 2010). However, it is interesting to note that PWS-IC*^del^* mice show a normal ghrelin response to fasting (Table 1) and re-feeding (unpublished data), as reported recently in humans with this condition (Kuppens *et al*. 2016).

It is also possible that proportionate hyperphagia may arise from impairment of the anorexic oxytocin circuitry. Although we have yet to quantify oxytocin expression in PWS-IC*^del^* mice, a reduction in the population of parvocellular oxytocin neurons has previously been described in humans with PWS (Swaab *et al*. 1995) and in mice with a specific deletion of *Magel2* (Schaller *et al*. 2010). Indeed, postnatal injections of oxytocin have been reported to restore suckling in *Magel2*-null mice, preventing neonatal mortality (Schaller *et al*. 2010) and ameliorating deficits in social and learning behaviour in adults (Meziane *et al*. 2015). As *Magel2* expression is absent in PWS-IC*^del^* mice (Chamberlain *et al*. 2004, Relkovic *et al*. 2010), it is likely that correcting any imbalance in the oxytocin system in these animals may have similar beneficial effects, but whether this will prevent hyperphagia in adults and restore fat deposition remains to be determined.

In the context of elevated proportionate caloric intake (which was seen despite correcting for hoarding behaviour), it is possible that leanness occurs in PWS-IC*^del^* mice as a result of impaired nutrient absorption. For the dietary lipids at least this does not appear to be the case, as circulating sterol esters, which reflect dietary lipid absorption (Jones & Glomset 1985), are unaffected (Table 1). In addition, although lipid processing in the liver may be disturbed (Fig. 1), the absence of any significant reduction in circulating TAG (Table 1) indicates that hepatic lipid output was not significantly curtailed.

The normal circulating lipid profile in the context of proportionate hyperphagia implies an increase in substrate utilisation. However, we have previously reported that locomotor activity in PWS-IC*^del^* mice is significantly reduced (Doe *et al*. 2009, Relkovic *et al*. 2010). We therefore examined whether the energy utilisation associated with BAT-mediated thermogenesis was elevated in this model. This appears to be the case. The halving of isBAT weight, which reflects reduced TAG storage, together with the increased transdifferentiation of white adipocytes to ‘beige’ or ‘brite’ adipocytes, is consistent with an increased demand for heat production (Barbatelli *et al*. 2010, Cinti 2011, Schultz *et al*. 2013). Similarly, the increased surface temperature in the head and interscapular regions (directly above the isBAT) in PWS-IC*^del^* mice indicates an elevation in non-shivering thermogenesis. In this context, it should be noted that although reduced locomotor activity in PWS-IC*^del^* mice may increase the infra-red signal measured, this does not result in an increase in surface temperature in those regions not associated with BAT activity, or in radiant temperature, which would be particularly susceptible to a reduction in movement. Thus, despite an absence of increased UCP1 expression, the classic marker of thermogenic activity, in PWS-IC*^del^* mice (Fig. 3B), our data indicate that lipid utilisation in BAT is elevated in this model.

Evidence of elevated thermogenesis in PWS is sketchy, but our data extend previous reports that *Snord116del* mice show elevated daytime subcutaneous temperature (Lassi *et al*. 2016) and an improved ability to maintain core body temperature on cold exposure (Ding *et al*. 2008). In humans, a small but statistically significant proportion of PWS children are reported to experience persistent (Ince *et al*. 2005) or episodic hypothermia (surface temperature <94°F) (Williams *et al*. 1994), whereas fingertip temperature is unaltered (DiMario & Burleson 2002). As BAT is now emerging as a potentially important tissue in adult humans, particularly in the context of obesity (Cannon & Nedergaard 2010, Cinti 2011), a comprehensive analysis of BAT function in individuals with PWS is now required.

Elevated thermogenesis in PWS-IC*^del^* mice is potentially significant because elevating BAT activity is highly effective in sequestering circulating lipid (Bartelt *et al*. 2011), thereby ‘starving’ WAT of energy substrate. When compounded by the increased energy utilisation of the more abundant ‘beige’/‘brite’ adipocytes within the WAT stores, this could result in the intra-abdominal leanness observed. To test this hypothesis, we examined the adiposity in PWS-IC*^del^* mice after suppressing thermogenic activity in a thermoneutral environment. Although the thermoneutral set point may be higher in smaller PWS-IC*^del^* mice (due to their increased surface area to volume ratio (Speakman & Keijer 2012)), maintaining mice at 30°C was clearly effective in suppressing thermogenesis, BAT mass increasing in WT and in PWS-IC*^del^* males (Fig. 3A). However, this failed to induce a corresponding elevation in WAT mass, body weight gain only being elevated transiently in WT females. This surprising outcome is most likely due to the influence of ambient temperature on food intake, proportionate hyperphagia in PWS-IC*^del^* males being abolished at thermoneutrality. Although this may suggest that simple elevations in ambient temperature may reduce orexigenic drive in human PWS, it demonstrates that elevated thermogenesis is not the primary cause of abdominal leanness in PWS-IC*^del^* mice.

In the absence of reduced energy intake/absorption or a causal increase in energy expenditure, leanness may result from a primary failure of lipid storage in WAT. Storage capacity is determined by adipogenesis (the terminal differentiation of pre-adipocytes), whereas lipid accumulates in response to increased substrate supply/uptake, *de novo* lipogenesis and/or decreased lipolysis or lipid export, resulting in hypertrophy (Wells 2009).

We did not quantify intra-abdominal adipogenesis directly, but the reduced adiposity in the tibial marrow of PWS-IC*^del^* mice was partly due to a 50% reduction in adipocyte number (data not shown). Evidence of reduced adipogenesis in subcutaneous WAT has recently been reported in human PWS, adipocyte progenitor number being halved (Cadoudal *et al*. 2014). These findings are consistent with a reduction in circulating GH, as GH promotes pre-adipocyte differentiation (Guller *et al*. 1989), and GH replacement in children with PWS restores pre-adipocyte numbers (Cadoudal *et al*. 2014).

Despite a reduction in lipid storage capacity, humans with PWS still develop obesity, whereas in PWS-IC*^del^* mice, intra-abdominal adipocyte size is reduced by 69% (Fig. 1E). With lipolysis and lipid export apparently unaffected (as indicated by normal circulating FFAs and normal lipolytic responses in cultured human adipocytes (Cadoudal *et al*. 2014)), the only option remaining is a failure of lipid uptake and/or synthesis. To test this, we monitored weight gain in mice maintained on standard and high-fat diets. Although we acknowledge the limitation of short time periods used, it is remarkable that despite increased caloric intake on the high-fat diet, PWS-IC*^del^* males, unlike WT littermates, failed to gain weight. When taken together with the reduced adipocyte size and the absence of elevated WAT mass at thermoneutrality, this implies that PWS-IC*^del^* adipocytes fail to import energy substrate.

**Figure 5:**
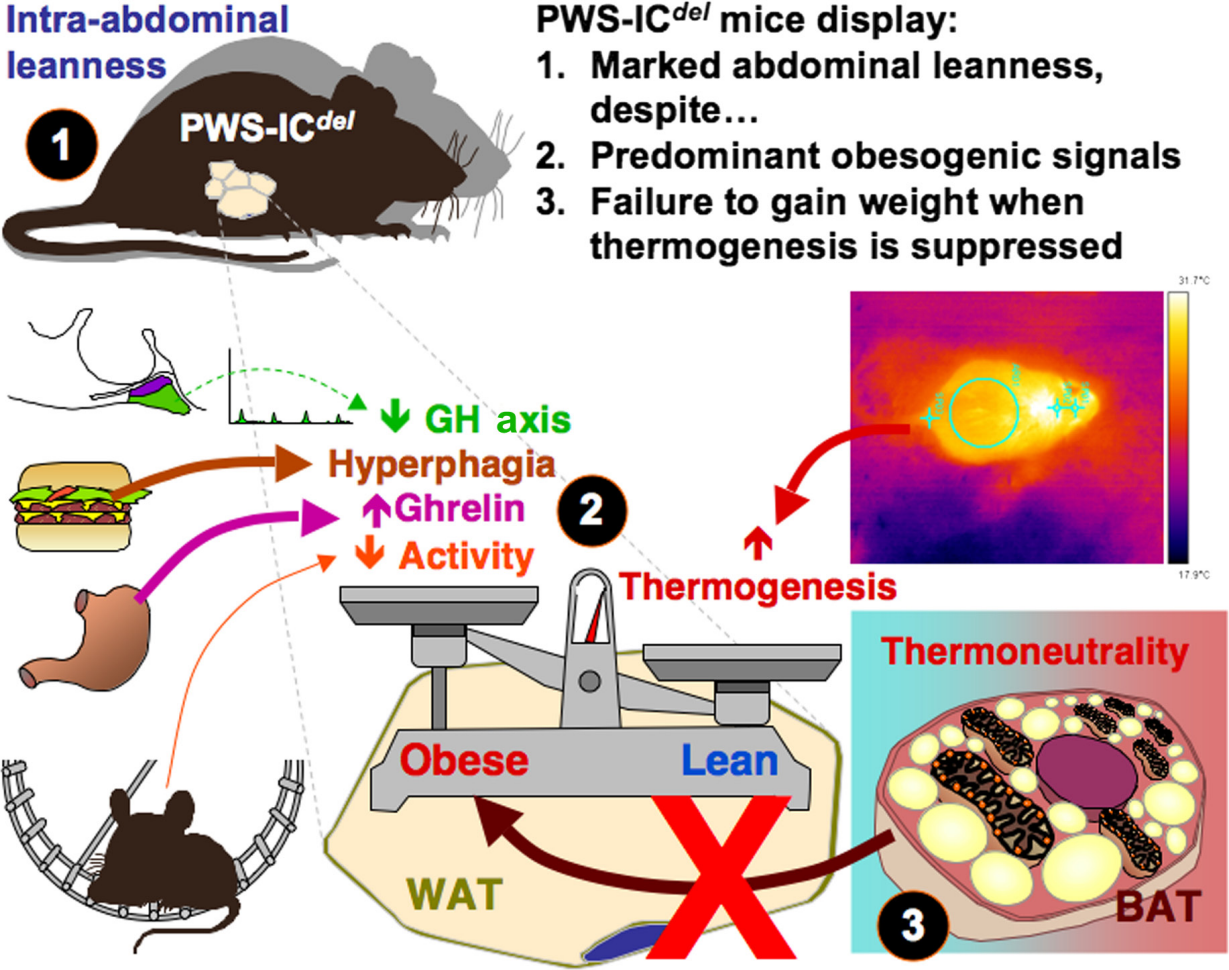
Mechanisms of leanness in PWS-IC*^del^* mice. PWS-IC*^del^* mice display marked abdominal leanness (1; current study) despite a preponderance of obesogenic signals (underactive GH axis (current study), proportionate hyperphagia (with pronounced food hoarding) (current study; Davies *et al*. 2015), hyperghrelinaemia (current study; Davies *et al*. 2015) and reduced physical activity (Doe *et al*. 2009, Relkovic *et al*. 2010)) (2). Suppression of elevated thermogenesis (current study) in brown adipose tissue (BAT) by maintenance in a thermoneutral environment (3) fails to elevate white adipose tissue (WAT) mass (current study). A full colour version of this figure is available at http://dx.doi.org/10.1530/JOE-16-0367.

In summary, when considered alongside our previous studies, the current data indicate that PWS-IC*^del^* mice display many of the endocrine and metabolic characteristics of human PWS, including marked hyperghrelinaemia and proportionate hyperphagia (Fig. 5). However, despite these obesogenic influences (Thompson *et al*. 2004, Wells 2009, Overton 2010), PWS-IC*^del^* mice show profound intra-abdominal leanness. Although this leanness may arise in part from a reduction in lipid storage capacity, the primary cause appears to be a failure of lipid accumulation in white adipocytes. Intra-abdominal leanness may result in elevated food hoarding and a compensatory increase in thermogenesis. Given our demonstration that the obesogenic environment of PWS may be overcome, determination of the functionality of parallel mechanisms in mice and humans may help to alleviate the morbid obesity usually present in children with this condition.

## Supplementary data

This is linked to the online version of the paper at http://dx.doi.org/10.1530/JOE-16-0367.

## Declaration of interest

The authors declare that there is no conflict of interest that could be perceived as prejudicing the impartiality of the research reported.

## Funding

This works was supported a Research Council UK Dorothy Hodgkin Postgraduate Award in collaboration with GlaxoSmithKline (to D R); the Prader–Willi Syndrome Association UK (to A R I and T W), Biotechnology and Biological Sciences Research Council (to A R I; BB/J016756/1) and Foundation for Prader–Willi Research (to T W); and Seed Corn Funding (Schools of Biosciences and the MRC Centre for Neuropsychiatric Genetics and Genomics, Cardiff University) (to T W and A R I).

## References

[bib1] Barbatelli G Murano I Madsen L Hao Q Jimenez M Kristiansen K Giacobino JP De Matteis R Cinti S 2010 The emergence of cold-induced brown adipocytes in mouse white fat depots is determined predominantly by brown adipocyte transdifferentiation. American Journal of Physiology: Endocrinology and Metabolism 298 E1244–E1253. (10.1152/ajpendo.00600.2009) 20354155

[bib2] Bartelt A Bruns OT Reimer R Hohenberg H Ittrich H Peldschus K Kaul MG Tromsdorf UI Weller H Waurisch C 2011 Brown adipose tissue activity controls triglyceride clearance. Nature Medicine 17 200–205. (10.1038/nm.2297) 21258337

[bib3] Bervini S Herzog H 2013 Mouse models of Prader-Willi syndrome: a systematic review. Frontiers in Neuroendocrinology 34 107–119. (10.1016/j.yfrne.2013.01.002) 23391702

[bib4] Bischof JM Stewart CL Wevrick R 2007 Inactivation of the mouse *Magel2* gene results in growth abnormalities similar to Prader-Willi syndrome. Human Molecular Genetics 16 2713–2719. (10.1093/hmg/ddm225) 17728320

[bib5] Bizzari C Rigamonti AE Luce A Cappa M Cella SG Berini J Sartorio A Müller EE Salvatoni A 2010 Children with Prader-Willi syndrome exhibit more evident meal-induced responses in plasma ghrelin and peptide YY levels than obese and lean children. European Journal of Endocrinology 162 499–505. (10.1530/EJE-09-1033) 20019130

[bib6] Butler M 1990 Prader-Willi syndrome: current understanding of cause and diagnosis. American Journal of Medical Genetics 35 319–332. (10.1002/ajmg.1320350306) 2309779 PMC5493042

[bib7] Cadoudal T Buléon M Segenès C Diene G Desneulin F Molinas C Eddiry S Conte-Auriol F Daviaud D Martin PG 2014 Impairment of adipose tissue in Prader-Willi syndrome rescued by growth hormone treatment. International Journal of Obesity 38 1234–1240. (10.1038/ijo.2014.3) 24406482

[bib8] Cannon B Nedergaard J 2010 Metabolic consequences of the presence or absence of the thermogenic capacity of brown adipose tissue in mice (and probably humans). International Journal of Obesity 34 S7–S16. (10.1038/ijo.2010.177) 20935668

[bib9] Chamberlain SJ Johnstone KA DuBose AJ Bartolomei MS Resnick JL Brannan CI 2004 Evidence for genetic modifiers of postnatal lethality in PWS-IC deletion mice. Human Molecular Genetics 13 2971–2977. (10.1093/hmg/ddh314) 15459179

[bib10] Cinti S 2011 Between brown and white: novel aspects of adipocyte differentiation. Annals of Medicine 43 104–115. (10.3109/07853890.2010.535557) 21254898

[bib11] Cummings DE Clement K Purnell JQ Vaisse C Foster KE Frayo RS Schwartz MW Basdevant A Weigle DS 2002 Elevated plasma ghrelin levels in Prader-Willi syndrome. Nature Medicine 8 643–644. (10.1038/nm0702-643) 12091883

[bib12] Dailey ME Bartness TJ 2008 Fat pad-specific effects of lipectomy on foraging, food hoarding, and food intake. American Journal of Physiology: Regulatory, Integrative and Comparative Physiology 294 R321–R328. (10.1152/ajpregu.00230.2007) PMC3509276 18003790

[bib13] Davies JR Humby T Dwyer DM Garfield AS Furby H Wilkinson AL Wells T Isles AR 2015 Calorie seeking, but no hedonic response, contributes to hyperphagia in a mouse model for Prader-WIlli Syndrome. European Journal of Neuroscience 42 2105–2113. (10.1111/ejn.12972) 26040449 PMC4949663

[bib14] Davies JS Kotokorpi P Eccles SR Barnes SK Tokarczuk PF Allen SK Whitworth HS Guschina IA Evans BA Mode A 2009 Ghrelin induces abdominal obesity via GHS-R-dependent lipid retention. Molecular Endocrinology 23 914–924. (10.1210/me.2008-0432) 19299444 PMC2691683

[bib15] Deacon RMJ 2006 Assessing hoarding in mice. Nature Protocols 1 2828–2830. (10.1038/nprot.2006.171) 17406541

[bib16] DelParigi A Tschöp M Heiman ML Salbe AD Vozarova B Sell SM Bunt JC Tataranni PA 2002 High circulating ghrelin: a potential cause for hyperphagia and obesity in Prader-Willi syndrome. Journal of Clinical Endocrinology and Metabolism 87 5461–5464. (10.1210/jc.2002-020871) 12466337

[bib17] DiMario FJ Jr Burleson JA 2002 Cutaneous blood flow and thermoregulation in Prader-Willi syndrome patients. Pediatric Neurology 26 130–133. (10.1016/S0887-8994(01)00386-1) 11897477

[bib18] Ding F Li HH Zhang S Solomon NM Camper SA Cohen P Francke U 2008 SnoRNA Snord116 (Pwcr1/MBII-85) deletion causes growth deficiency and hyperphagia in mice. PLoS ONE 3 e1709 (10.1371/journal.pone.0001709) 18320030 PMC2248623

[bib19] Doe CM Relkovic D Garfield AS Dalley JW Theobald DE Humby T Wilkinson LS Isles AR 2009 Loss of the imprinted snoRNA mbii-52 leads to increased 5htr2c pre-RNA editing and altered 5HT2CR-mediated behaviour. Human Molecular Genetics 18 2140–2148. (10.1093/hmg/ddp137) 19304781 PMC2685753

[bib20] Erdie-Lalena CR Holm VA Kelly PC Frayo RS Cummings DE 2006 Ghrelin levels in young children with Prader-Willi syndrome. Journal of Pediatrics 149 199–204. (10.1016/j.jpeds.2006.04.011) 16887433

[bib21] Ge Y Ohta T Driscoll DJ Nicholls RD Kalra SP 2002 Anorexigenic melanocort signalling in the hypothalamus is augmented in association with failure-to-thrive in a transgenic mouse model for Prader-Willi syndrome. Brain Research 957 42–45. (10.1016/S0006-8993(02)03583-7) 12443978

[bib22] Glad CA Kitchen EE Russ GC Harris SM Davies JS Gevers EF Gabrielsson BG Wells T 2011 Reverse feeding suppresses the activity of the GH axis and induces a preobesogenic state. Endocrinology 152 689–882. (10.1210/en.2010-0768) 21209022

[bib23] Goldstone AP Thomas EL Brynes AE Bell JD Frost G Saeed N Hajnal JV Holland A Bloom SR 2001 Visceral adipose tissue and metabolic complications of obesity are reduced in Prader-Willi syndrome female adults: evidence for novel influences on body fat distribution. Journal of Clinical Endocrinology and Metabolism 86 4330–4338. (10.1210/jcem.86.9.7814) 11549670

[bib24] Goldstone AP Thomas EL Brynes AE Castroman G Edwards R Ghatei MA Frost G Holland AJ Grossman AB Korbonits M 2004 Elevated fasting plasma ghrelin in Prader-Willi syndrome is not solely explained by their reduced visceral adiposity and insulin resistance. Journal of Clinical Endocrinology and Metabolism 89 1718–1726. (10.1210/jc.2003-031118) 15070936

[bib25] Guller S Sonenberg M Wu KY Szabo P Corin RE 1989 Growth hormone-dependent events in adipose differentiation of 3T3-F442A fibroblasts: modulation of macromolecular synthesis. Endocrinology 125 2360–2367. (10.1210/endo-125-5-2360) 2477229

[bib26] Haqq AM Farooqi IS O’Rahilly S Stadler DD Rosenfeld RG Pratt KL LaFranchi SH Purnell JQ 2003 Serum ghrelin levels are inversely correlated with body mass index, age, ad insulin concentrations in normal children and are markedly increased in Prader-Willi syndrome. Journal of Clinical Endocrinology and Metabolism 88 174–178. (10.1210/jc.2002-021052) 12519848

[bib27] Ince E Çiftçi E Tekin M Kendirli T Tutar E Dalgiç S Öncel S Dogru Ü 2005 Characteristics of hyperthermia and its complications in patients with Prader Willi syndrome. Pediatrics International 47 550–553. (10.1111/j.1442-200x.2005.02124.x) 16190963

[bib28] Jones A Glomset J 1985 Chapter 4 Biosynthesis, function and metabolism of sterol esters. New Comprehensive Biochemistry 12 95–119. (10.1016/s0167-7306(08)60680-8)

[bib29] Kates M 1986 Techniques in Lipidology, 2nd ed. Amsterdam, The Netherlands: Elsevier.

[bib30] Keen-Rhinehart E Bartness TJ 2005 Peripheral ghrelin injections stimulate food intake, foraging and food hoarding in Siberian hamsters. American Journal of Physiology: Regulatory, Integrative and Comparative Physiology 288 R716–R722. (10.1152/ajpregu.00705.2004) 15576659

[bib31] Keen-Rinehart E Dailey MJ Bartness T 2010 Physiological mechanisms for food-hoarding motivation in animals. Philosophical Transactions of the Royal Society B 365 961–975. (10.1098/rstb.2009.0225) PMC2830250 20156819

[bib32] Kozlov SV Bogenpohl JW Howell MP Wevrick R Panda S Hogenesch JB Muglia LJ Van Gelder RN Herzog ED Stewart CL 2007 The imprinted gene Magel2 regulates normal circadian output. Nature Genetics 39 1266–1272. (10.1038/ng2114) 17893678

[bib33] Kuppens RJ Delhanty PJ Huisman TM van der Lely AJ Hokken-Koelega AC 2016 Acylated and unacylated ghrelin during OGTT in Prader-Willi syndrome: support for normal response to food intake. Clinical Endocrinology 85 488–494. (10.1111/cen.13036) 26850227

[bib34] Lassi G Priano L Maggi S Garcia-Garcia C Balzani E El-Assawy N Pagani M Tinarelli F Giardino D Mauro A 2016 Deletion of the *Snord116/SNORD116* alters sleep in mice and patients with Prader-Willi syndrome. Sleep 39 637–644. (10.5665/sleep.5542) 26446116 PMC4763347

[bib35] Lawrence CB Snape AC Baudoin FM Luckman SM 2002 Acute central ghrelin and GH secretagogues induce feeding and activate appetite centers. Endocrinology 143 155–162. (10.1210/endo.143.1.8561) 11751604

[bib36] Meziane H Schaller F Bauer S Villard C Matarazzo V Riet F Guillon G Lafitte D Desarmenien MG Tauber M 2015 An early postnatal oxytocin treatment prevents social and learning deficits in adult mice deficient in *Magel2*, a gene involved in Parder-Willi sundrome. Biological Psychiatry 78 85–94. (10.1016/j.biopsych.2014.11.010) 25599930

[bib37] Nakazato M Murakami N Date Y Kojima M Matsuo H Kangaw K Matsukura S 2001 A role for ghrelin in the central regulation of feeding. Nature 409 194–198. (10.1038/35051587) 11196643

[bib38] Overton JM 2010 Phenotyping small animals as models for the human metabolic syndrome: thermoneutrality matters. International Journal of Obesity 34 S53–S58. (10.1038/ijo.2010.240) 21151148

[bib39] Peters J 2008 Prader-Willi and snoRNAs. Nature Genetics 40 688–689. (10.1038/ng0608-688) 18509309

[bib40] Qi Y Purtell L Fu M Lee MJ Aepler J Zhang L Loh K Enriquez RF Baldock PA Zolotukhin S 2016 Snord116 is critical in the regulation of food intake and body weight. Scientific Reports 6 18614 (10.1038/srep18614) 26726071 PMC4698587

[bib41] Relkovic D Doe CM Humby T Johnstone KA Resnick JL Holland AJ Hagan JJ Wilkinson LS Isles AR 2010 Behavioural and cognitive abnormalities in an imprinting centre deletion mouse model for Prader-Willi syndrome. European Journal of Neuroscience 31 156–164. (10.1111/j.1460-9568.2009.07048.x) 20092561

[bib42] Sahoo T del Gaudio D German JR Shinawi M Peters SU Person RE Garnica A Cheung SW Beaudet AL 2008 Prader-Willi phenotype caused by deficiency for the HBII-85 C/D box small nucleolar RNA cluster. Nature Genetics 40 719–721. (10.1038/ng.158) 18500341 PMC2705197

[bib43] Schaller F Watrin F Sturny R Massacrier A Szepetowski P Muscatelli F 2010 A single postnatal injection of oxytocin rescues the lethal feeding behaviour in mouse newborns deficient for the imprinted Magel2 gene. Human Molecular Genetics 19 4895–4905. (10.1093/hmg/ddq424) 20876615

[bib44] Schultz TJ Huang P Huang TL Xue R McDougall LE Townsend KL Cypess AM Mishina Y Gussoni E Tseng Y 2013 Brown-fat paucity due to impaired BMP signalling induces compensatory browning of white fat. Nature 495 379–383. (10.1038/nature11943) 23485971 PMC3623555

[bib45] Speakman JR Keijer J 2012 Not so hot: optimal housing temperatures for mice to mimic the thermal environment of humans. Molecular Metabolism 21 5–9. (10.1016/j.molmet.2012.10.002) PMC3757658 24024125

[bib46] Stefan M Ji H Simmons RA Cummings DE Ahima RS Friedman MI Nicholls RD 2005 Hormonal and metabolic defects in a prader-willi syndrome mouse model with neonatal failure to thrive. Endocrinology 146 4377–4385. (10.1210/en.2005-0371) 16002520

[bib47] Swaab DF Purba JS Hofman MA 1995 Alterations in the hypothalamic parventricular nucleus and its oxytocin neurons (putative satiety cells) in Prader-Willi syndrome: a study of five cases. Journal of Clinical Endocrinology and Metabolism 80 573–579. (10.1210/jcem.80.2.7852523) 7852523

[bib48] Thompson NM Gill DA Davies R Loveridge N Houston PA Robinson ICAF Wells T 2004 Ghrelin and des-octanoyl ghrelin promote adipogenesis directly in-vivo by a mechanism independent of the type 1a growth hormone secretagogue receptor. Endocrinology 145 234–242. (10.1210/en.2003-0899) 14551228

[bib49] Wells T 2009 Ghrelin – defender of fat. Progress in Lipid Research 48 257–274. (10.1016/j.plipres.2009.04.002) 19409927

[bib50] Wells T Davies JR Guschina IA Ball DJ Davies JS Davies VJ Evans BAJ Votruba M 2012 Opa3, a novel regulator of mitochondrial function, controls thermogenesis and abdominal fat mass in a mouse model for Costeff syndrome. Human Molecular Genetics 21 4836–4844. (10.1093/hmg/dds315) 22869679

[bib51] Williams MS Rooney BL Williams J Josephson K Pauli R 1994 Investigation of thermoregulatory characteristics in patients with Prader-Willi syndrome. American Journal of Medical Genetics 49 302–307. (10.1002/ajmg.1320490312) 8209890

[bib52] Yang T Adamson TE Resnick JL Leff S Wevrick R Francke U Jenkins NA Copeland NG Brannan CI 1998 A mouse model for Prader-Willi syndrome imprinting-centre mutations. Nature Genetics 19 25–31. (10.1038/ng0598-25) 9590284

